# Benchmarking deep learning methods for C_*α*_ atom prediction in cryo-EM density maps

**DOI:** 10.1093/bioinformatics/btag350

**Published:** 2026-06-02

**Authors:** Tian Zhang, Zhe Liu, Yiqing Ma, Chenjie Feng, Renmin Han

**Affiliations:** Research Center for Mathematics and Interdisciplinary Sciences; Cheeloo College of Medicine, Qilu Hospital (Qingdao), Shandong University, Qingdao 266237, China; College of Medical Information and Engineering, Ningxia Medical University, Yinchuan 750004, China; Research Center for Mathematics and Interdisciplinary Sciences; Cheeloo College of Medicine, Qilu Hospital (Qingdao), Shandong University, Qingdao 266237, China; College of Medical Information and Engineering, Ningxia Medical University, Yinchuan 750004, China; Research Center for Mathematics and Interdisciplinary Sciences; Cheeloo College of Medicine, Qilu Hospital (Qingdao), Shandong University, Qingdao 266237, China; College of Medical Information and Engineering, Ningxia Medical University, Yinchuan 750004, China; College of Medical Information and Engineering, Ningxia Medical University, Yinchuan 750004, China; Research Center for Mathematics and Interdisciplinary Sciences; Cheeloo College of Medicine, Qilu Hospital (Qingdao), Shandong University, Qingdao 266237, China

## Abstract

**Motivation:**

With the advancement of cryo-electron microscopy (cryo-EM) into the atomic resolution era, accurate Cα atom modeling has become essential for macromolecular structure determination. However, existing evaluation systems overly rely on full-atom metrics and lack a dedicated, comprehensive benchmark for assessing Cα prediction modules within automated modeling tools.

**Results:**

To address this gap, we establish a rigorous benchmark to evaluate the Cα prediction performance of four prominent deep learning-based methods (ModelAngelo, DeepMainMast, EModelX, and CryoAtom) across multiple dimensions. We construct a diverse dataset covering a wide range of resolutions (1–8 Å), molecular weights, and noise levels. A novel evaluation framework is introduced, incorporating multi-threshold RMSD-based metrics (1–3 Å) alongside advanced point-cloud similarity measures (Chamfer Distance, Earth Mover’s Distance) for quantitative and nuanced assessment. Our results reveal that method performance is highly dependent on the chosen evaluation criteria and intrinsic data characteristics. ModelAngelo excels under loose thresholds with high-quality data but shows sensitivity to resolution degradation; CryoAtom demonstrates notable computational efficiency, however, its completeness-oriented design leads to a certain loss of precision; EModelX demonstrates balanced generalization across varied conditions; DeepMainMast achieves high localization accuracy under stringent criteria but incurs a high computational cost.

**Availability and implementation:**

This work provides a reproducible, Cα-centric evaluation framework to guide method development and advance automated cryo-EM structure determination. The source code for the benchmark and evaluation metrics is freely available at https://github.com/zhtianz/Benchmarking\_CA.

## 1 Introduction

The precise determination of 3D protein structures is crucial for understanding their biological functions and facilitating rational drug design (Schmidt *et al.* 2014). Although X-ray crystallography has long been regarded as the gold standard for high-resolution structure determination [with approximately 85% of Protein Data Bank (PDB) entries exhibiting resolution better than 2 Å], recent revolutionary advances in cryo-electron microscopy (cryo-EM) technology, particularly since the widespread adoption of direct electron detectors in 2015, have substantially expanded the scope of structural biology (Kühlbrandt 2014, Nakane *et al.* 2020, Gao *et al.* 2021). Cryo-EM offers distinct advantages in resolving large macromolecular complexes and capturing conformational heterogeneity. Along with its rapidly increasing data throughput, the number of entries in the Electron Microscopy Data Bank (EMDB) has grown exponentially, increasing from approximately 3000 in 2015 to >40 000 by 2025, of which around 20% reach resolutions between 3 and 5 Å. This rapid growth marks cryo-EM’s progressive transition into the atomic-resolution era (Su *et al.* 2021).

In high-quality, high-resolution EM density maps (<5 Å), the protein backbone structure can be distinguished. The protein backbone is defined as a continuous chain of atoms that runs along the entire length of the protein (Domanska *et al.* 2019). The backbone particular consists of the nitrogen atom (N), the alpha-carbon atom (Cα), and the carbonyl carbon atom (C). Among these three atoms, the α-carbon (Cα) is of particularly importance, as it serves as the central reference point of each amino acid residue within the protein (Martynowycz *et al.* 2019). Therefore, accurately predicting the position of each Cα atom along the backbone facilitates the identification of individual amino acid residues within the overall protein structure (Zhu *et al.* 2018). In this context, accurate modeling of the Cα backbone, as a critical step in structure determination, has become increasingly prominent. It not only provides a foundational scaffold for functional site analysis but also serves as a key starting point for exploring protein flexibility and dynamic regions.

Despite significant progress, achieving fully automated and high-precision Cα modeling from cryo-electron microscopy (cryo-EM) density maps remains challenging, particularly in medium-to-low-resolution regions or areas with low signal-to-noise ratios (Wang and Sigworth 2006, Bai *et al.* 2015, Nogales and Scheres 2015, Merk *et al.* 2016). The introduction of deep learning has substantially enhanced the efficiency and automation of structure modeling, leading to the development of integrated tools such as ModelAngelo (Jamali *et al.* 2024), DeepMainMast (Terashi *et al.* 2024), EModelX (Chen *et al.* 2024), and CryoAtom (Su *et al.* 2026).

Current mainstream methods generally adopt a two-step workflow: first predicting Cα atomic positions and topology from density signals, and then constructing full-atomic models through sequence assignment and matching. However, the Cα modeling modules of these tools use distinct network architectures and post-processing methods (detailed in Table 1), leading to considerable performance variability across different data conditions (Si *et al.* 2020). This variability arises from differences in algorithmic design, limitations in the representativeness of training datasets, and insufficient robustness to heterogeneity in density quality. The deep learning-based pipelines for Cα prediction typically follow a three-stage process: feature extraction, clustering, and geometric optimization. While all four evaluated tools adhere to this general framework, they exhibit distinct implementations that lead to varied outcomes. For instance, ModelAngelo uses an FPN-based CNN with dual-channel input (He *et al.* 2016, Lin *et al.* 2017), DeepMainMast utilizes a UNet3+ architecture (Huang *et al.* 2020), EModelX adopts a multi-task 3D residual U-Net (Lee *et al.* 2017), and CryoAtom centers on a 3D convolutional U-Net followed by clustering steps like MeanShift (Ronneberger *et al.* 2015, Terashi and Kihara 2018, Chen *et al.* 2023, Wang *et al.* 2023).

**Table 1:** Summary of evaluated methods with key characteristics and resources.

Methods	Architecture	Cluster method	Training dataset Size	Training resolution range	Year	Link
ModelAngelo	CNN	MeanShift	3715	<4 Å	2024	https://github.com/3dem/model-angelo
DeepMainMast	Unet3+	MeanShift	197	2.5–5 Å	2023	https://github.com/kiharalab/DeepMainMast
EModelX	3D Residual U-Net	DBSCAN	1529	2–4 Å	2024	https://bio-web1.nscc-gz.cn/app/EModelX
CryoAtom	3D Convolutional U-Net	MeanShift	5731	<4 Å	2024	https://github.com/SBQ-1999/CryoAtom

Beyond network architecture, the post-processing clustering strategy is a key differentiator. The evaluated tools primarily use two algorithms: MeanShift and Density-Based Spatial Clustering of Applications with Noise (DBSCAN). MeanShift is a centroid-based method that iteratively shifts candidate points toward regions of highest data density, effectively discovering clusters without requiring a pre-defined cluster count. This makes it robust for datasets with an unknown number of Cα atoms. In contrast, DBSCAN forms clusters by connecting densely packed points, explicitly classifying sparser outliers as noise. This property can be advantageous for distinguishing true signal from background in low-density regions but introduces sensitivity to its distance and minimum sample parameters. The choice of clustering algorithm thus directly affects the sensitivity, specificity, and noise resilience of the final Cα trace.

However, existing evaluation systems largely emphasize the overall quality of final full-atomic models, while lacking a dedicated and fundamental assessment of the initial Cα modeling stage. In particular, systematic comparisons of Cα prediction accuracy, consistency, and robustness across different resolution ranges, signal-to-noise ratio conditions, and conformational variability remain scarce (Si *et al.* 2020, Giri and Cheng 2023). This evaluation gap is compounded by multiple factors: an overemphasis on full-atom models, reliance on uniform noise simulations rather than authentic cryo-EM data characteristics, and the use of oversimplified metrics with inconsistent preprocessing pipelines. Consequently, it becomes difficult for researchers to select appropriate tools tailored to specific data characteristics, and the identification of clear directions for algorithmic improvement is hampered.

Here, we present a systematic evaluation of the Cα atom prediction modules in four mainstream automated modeling tools, namely ModelAngelo, DeepMainMast, EModelX, and CryoAtom. First, by substituting the native Cα prediction components of these modeling tools, we demonstrate that high-accuracy Cα atom prediction leads to measurable improvements in subsequent overall structure reconstruction. Next, by constructing a benchmark dataset spanning diverse resolutions, molecular weights, and noise conditions, we conduct quantitative analyses from the perspectives of spatial accuracy, global similarity, and local similarity structural consistency. Through this comprehensive evaluation, this study aims to provide the scientific community with a rigorous and objective performance reference, thereby facilitating further optimization of this critical component in cryo-EM structure determination pipeline.

## 2 Materials and methods

**Figure 1:**
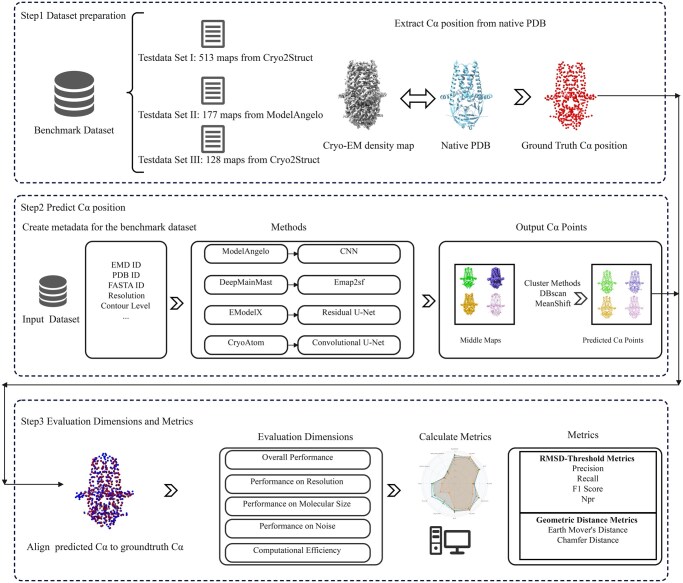
Overview of our benchmarking workflow. Step 1 Data Preparation: Extract Cα atoms from PDB structures corresponding to density maps as ground truth. α Step 2 Cα Atom Prediction: Four software tools predict Cα atoms; intermediate density maps from network models are clustered to obtain predicted Cα atoms. Step 3 Metric Calculation: Compare predicted Cα atoms with ground truth using RMSD-Threshold Metrics and Geometric Distance Metrics under five scenarios to evaluate accuracy and adaptability.

This chapter delineates the methodological framework established for the present benchmarking study. The framework is architected around three principal components: (i) a unified pipeline for software execution and evaluation; (ii) the protocols governing the construction and preprocessing of the benchmark dataset. [Our benchmark dataset was constructed by amalgamating three publicly available collections of cryo-EM density maps: the complete 513-map test set from Cryo2Struct (Giri and Cheng 2024) (Testdata Set I); the 177-map test set from ModelAngelo (Jamali *et al.* 2024) (Testdata Set II) and the standard 128-map test set, also from Cryo2Struct (Giri and Cheng 2024) (Testdata Set III). The details of data processing and distribution are provided in Supplementary Material 1, available as supplementary data at *Bioinformatics* online, Fig. 1, available as supplementary data at *Bioinformatics* online, and Table 1, available as supplementary data at *Bioinformatics* online]; (iii) a suite of multidimensional metrics used for performance assessment. A primary objective of this design is to uphold fairness and ensure reproducibility in the evaluation, thereby yielding a comprehensive and reliable assessment report on the performance of automated Cα atom modeling for the scientific community.

### 2.1 Cα Atom prediction performance evaluation pipeline

To enable objective comparison of Cα atom prediction performance, we developed a specialized evaluation pipeline that isolates the core localization task from confounding variables in subsequent model-building procedures (Fig. 1).

All input data underwent standardized preprocessing, including resolution normalization (via low-pass filtering to 3.0–5.0 Å), SNR modulation through Gaussian noise addition (0.1–0.3 levels), and format conversion. This ensured uniform inputs across tools, eliminating preprocessing-related biases. A standardized post-processing pipeline intercepted Cα coordinate predictions at the network output stage rather than extracting them from final full-atom models. This approach isolates Cα localization accuracy from confounding factors introduced by downstream tasks like side-chain modeling or stereochemical refinement.

Performance was quantified using three complementary metric categories: (i) positional accuracy metrics (RMSD), (ii) detection capability metrics (precision, recall, F1-score), and (iii) global structural similarity metrics (Chamfer Distance, Earth Mover’s Distance). This framework comprehensively assesses atomic-level accuracy, detection efficacy, and topological integrity. Results were aggregated and stratified across key experimental dimensions resolution, SNR, and molecular weight to provide empirical guidance for tool selection under specific experimental conditions.

### 2.2 Evaluation metrics

The match-based metrics first establish correspondences between predicted and ground truth Cα atoms within a specified distance threshold δ, which is typically set to 3.0 Å to correspond with physiologically relevant distances such as typical hydrogen bond lengths. The core metrics are defined as follows, where P denotes the set of predicted Cα atoms and G denotes the set of ground truth Cα atoms.

Recall measures the completeness of detection.


(1) $$\text{Recall}=\frac{\left|\{g\in G:\min_{p\in P}||p-g||\leq \delta \}\right|}{\left|G\right|}$$

Precision evaluates the reliability of predictions.


(2) $$\text{Precision}=\frac{\left|\{p\in P:\min_{g\in G}||p-g||\leq \delta \}\right|}{\left|P\right|}$$

F1-score provides the harmonic mean of Precision and Recall.


(3) $$F_{1}=2\cdot \frac{\text{Precision}\cdot \text{Recall}}{\text{Precision}+\text{Recall}}$$

The Root Mean Square Deviation (RMSD) quantifies the local positional accuracy of the $N_{m}$ matched atom pairs. The metric is computed directly on the matched coordinates p and g without applying rotational or translational superposition, providing a measure of direct coordinate deviation in the original reference frame:


(4) $$\text{RMSD}=\sqrt{\frac{1}{N_{m}}\sum_{(p,g)\in M}{\left\|p-g\right\|}^{2}}$$

To assess localization accuracy at varying levels of stringency, RMSD is computed using three distinct matching thresholds (δ) for initial correspondence establishment: 3.0, 2.0, and 1.0 Å. The 3.0 Å threshold evaluates robust fold-level placement, 2.0 Å assesses high-accuracy backbone tracing, and the stringent 1.0 Å threshold serves as a limit test for near-atomic precision.

3 Å Cα Content assesses the spatial coverage of predictions relative to true atoms.


(5) $$3Å\text{ Content}=\frac{|\{g\in G:\min_{p\in P}||p-g||\leq 3.0Å\}|}{|G|}$$

Coverage quantifies the biological relevance of the predicted atoms.


(6) $$\text{Coverage}=\frac{\left|\{p\in P:\min_{g\in G}||p-g||\leq 3.0Å\}\right|}{\left|P\right|}$$

To quantify prediction completeness, we define a completeness index, Npr, as


(7) $$\text{Npr}=\frac{\mathrm{Npred}}{\mathrm{Nref}}$$

Here, Npred is the number of predicted Cα atoms and Nref is the number of Cα atoms in the reference structure. The reference structure is defined as the deposited native PDB model paired with each EMDB map in the benchmark dataset. An Npr>1 indicates that a method tends to produce a more complete trace than the deposited reference, whereas Npr<1 indicates a more conservative prediction. Importantly, Npr reflects completeness only and does not assess geometric correctness or density support of additional residues.

### 2.3 Point cloud similarity metrics

Recognizing that match-based metrics are inherently limited by a strict distance cutoff and can be insensitive to global structural errors, we complement our analysis with point cloud similarity metrics. These metrics treat the predicted and native structures as spatial distributions, thereby capturing global shape congruence without requiring explicit atomic pairing. Specifically, we use Chamfer Distance (CD) and Earth Mover’s Distance (EMD) to provide a more holistic measure of structural similarity.

Chamfer Distance (CD) measures how closely two point clouds cover each other. A low value indicates high overall shape similarity, and it is sensitive to outliers.


(8) $$\text{CD}(P,G)=\frac{1}{\left|P\right|}\sum_{p\in P}\min_{g\in G}\|p-g{\|}^{2}+\frac{1}{\left|G\right|}\sum_{g\in G}\min_{p\in P}\|g-p{\|}^{2}$$

Earth Mover’s Distance (EMD) (Wu *et al.* 2021) finds a global, optimal correspondence between points, measuring the minimum cost to transform one point cloud into the other. It effectively reveals global structural shifts.


(9) $$\text{EMD}(P,G)=\underset{\left\{f_{ij}\right\}}{\min}\sum_{i=1}^{m}\sum_{j=1}^{n}f_{ij}\cdot \|p_{i}-g_{j}{\|}_{2}$$

subject to: $f_{ij}\geq 0$, $\sum_{j}f_{ij}\leq 1$, $\sum_{i}f_{ij}\leq 1$, $\sum_{i,j}f_{ij}=\min(m,n)$.

By integrating atomic-level precision (Recall, Precision, RMSD), local matching quality (Content, Coverage), and global shape fidelity (CD, EMD), our framework delivers a comprehensive and nuanced evaluation of model performance.

### 2.4 Software and libraries

All computational analyses were performed using Python 3.8. The study utilized the following core scientific libraries: NumPy for fundamental array operations and numerical computations; SciPy for spatial data structures (specifically cKDTree), distance calculations (cdist, pdist), and combinatorial optimization (linear_sum_assignment); Biopython for reading and parsing atomic coordinate files in both PDB and mmCIF formats; and Matplotlib for generating all 2D and 3D visualizations presented in the manuscript. In addition, Q-score (Pintilie *et al.* 2020) was used to quantify the agreement between predicted Cα atoms and the cryo-EM density map, reflecting the local resolvability and map-fitting quality of the predicted coordinates.

## 3 Results

In this study, we present a comprehensive benchmark evaluation of four cutting-edge deep learning methods for Cα atom prediction in cryo-EM density maps: ModelAngelo, DeepMainMast, EModelX, and CryoAtom. The evaluation is structured to answer three fundamental questions: (i) How critical is accurate Cα prediction for subsequent all-atom modeling? (ii) How do these methods perform in terms of accuracy and efficiency under standardized benchmark conditions? (iii) How robust are they when confronted with real-world experimental variables such as varying resolutions, molecular weights, and noise levels? To this end, we first validate the foundational importance of precise Cα placement. We then conduct a systematic performance comparison across three public datasets, analyzing both predictive accuracy (using metrics such as precision, recall, F1-score, Chamfer Distance, and Earth Mover’s Distance at multiple RMSD thresholds) and computational speed. Finally, we perform an in-depth robustness analysis by re-evaluating the methods on data stratified by resolution and molecular weight, and on density maps perturbed with simulated Gaussian noise.

### 3.1 How critical is accurate Cα prediction for all-atom modeling?

To quantify the dependence of full-atom model accuracy on the initial Cα trace, we conducted a Cα replacement experiment on Testdata Set III. We compared models generated by CryoAtom and ModelAngelo under two conditions: using their own predicted Cα coordinates versus using the ground-truth Cα coordinates extracted from the native structures. The results, summarized in Table 2, demonstrate that providing ideal Cα coordinates substantially enhances the final model’s precision, confirming the foundational role of Cα prediction in the overall modeling pipeline.

**Table 2:** Improvement in all-atom model quality using native Cα atoms.^a^

Method	Improvement rate	Mean RMSD (Å)	*t*-statistic	*P*-value	Mean improvement
ModelAngelo	93.20%	1.088 → 1.049	−10.18	<0.0001	0.043 Å (3.6%)
CryoAtom	99.22%	1.170 → 1.023	−16.42	<0.0001	0.149 Å (12.5%)

a Comparison of all-atom model accuracy (3 Å RMSD) when using predicted Cα (left) versus native Cα atoms (right). Both methods show statistically significant improvement (paired *t*-test, p<0.0001) with native Cα atoms. CryoAtom demonstrates a higher improvement rate (99.22% versus 93.20%), greater RMSD reduction (0.149 Å versus 0.043 Å), and stronger statistical significance (t=−16.42 versus –10.18).

When supplied with native Cα coordinates, ModelAngelo’s average all-atom RMSD improved from 1.088 Å to 1.049 Å, a relative improvement of 3.6% (paired *t*-test, t=−10.18, *P*<.0001) with 93.20% of structures showing increased accuracy. The effect on CryoAtom was more pronounced: its average RMSD decreased from 1.170 Å to 1.023 Å, a 12.5% relative improvement (t=−16.42, *P*<.0001). This enhancement was observed in 99.22% of test cases, and the magnitude of RMSD reduction (0.149 Å) was over three times that of ModelAngelo (0.043 Å), indicating CryoAtom’s greater sensitivity to the quality of the input Cα trace.

**Figure 2:**
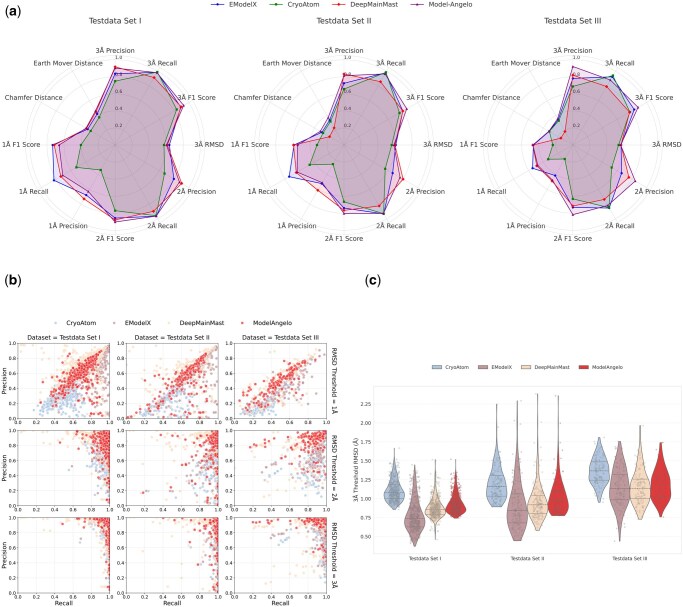
(a) Radar charts showing precision, recall, F1-score, CD (Chamfer Distance), and EMD (Earth Mover’s Distance) for datasets Testdata Set I, Testdata Set II, and Testdata Set III at 3, 2, and 1 Å RMSD thresholds. (b) Precision-recall balance scatter plots for datasets Testdata Set I, Testdata Set II, and Testdata Set III at 3, 2, and 1 Å RMSD thresholds. The *x*-axis represents recall rate, and the *y*-axis represents precision. (c) Violin plots of Cα RMSD within the 3 Å threshold. The *x*-axis represents three public datasets: Testdata Set I, Testdata Set II, and Testdata Set III. The *y*-axis shows the RMSD distribution of four software tools.

Similar improvements in structural fidelity were observed for both CryoAtom on EMD-26706 and ModelAngelo on EMD-22898 and EMD-26702 when native Cα coordinates were utilized (Details are provided in Supplementary Material 2.1, available as supplementary data at *Bioinformatics* online, Table 2, available as supplementary data at *Bioinformatics* online, Fig. 2, available as supplementary data at *Bioinformatics* online).

Collectively, these results establish that Cα positional accuracy is a key determinant of final all-atom model quality. The benchmark reveals both a strong positive correlation and method-specific sensitivity to Cα coordinate errors. This underscores that enhancing the precision of the Cα prediction module is a critical and effective strategy for advancing all-atom structure prediction methods.

It should be noted that EModelX and DeepMainMast were excluded from this experiment. Their architectures are designed such that the full-atom prediction stage is intrinsically dependent on additional information such as amino acid types information predicted concurrently during the Cα tracing step, rendering a Cα-only replacement infeasible.

### 3.2 Comparative benchmark of four methods on standard test datasets

#### 3.2.1 Performance of methods on public benchmark datasets

To comprehensively evaluate the performance of four Cα tracing programs (CryoAtom, EModelX, DeepMainMast, and ModelAngelo), we conducted a benchmark analysis on three distinct datasets: Testdata Set I, Testdata Set II, and Testdata Set III. The evaluation used multiple metrics, including precision, recall, F1-score, Chamfer Distance (CD), and Earth Mover’s Distance (EMD), under varying RMSD thresholds (1, 2, and 3 Å) to assess both accuracy and structural fidelity.

The radar charts in Fig. 2a provide a comprehensive comparison across all metrics. Under the relaxed 3 Å threshold, ModelAngelo shows the best overall performance on most metrics, particularly excelling in F1-score and recall on Testdata Set III while maintaining favorable CD and EMD metrics. CryoAtom demonstrates relatively stable recall under the 3 Å threshold but exhibits significant fluctuations at the stricter 1 Å threshold, indicating inconsistencies in local geometry reconstruction despite reliable backbone placement. EModelX and DeepMainMast show moderate overall performance, with DeepMainMast’s CD and EMD performance on Testdata Sets II and III being relatively poor, suggesting weaker ability to capture local structural features.

The precision-recall scatter plots (Fig. 2b) reveal important trade-offs between these complementary metrics. At the 3 Å threshold, all tools show improved performance, with points clustering toward the upper-right quadrant, indicating better overall prediction completeness. However, at the stringent 1 Å threshold, performance degrades substantially, with most points shifting to the lower-left quadrant. ModelAngelo consistently maintains positions closest to the upper-right corner across thresholds, particularly on Testdata Set III, indicating both high precision and recall. CryoAtom shows the highest recall values but with variable precision, especially evident at intermediate thresholds where its performance shows greater dispersion.

Analysis of RMSD distributions within the 3 Å threshold (Fig. 2c) provides further insights into prediction consistency. EModelX and DeepMainMast exhibit the tightest RMSD distributions centered around lower values (median approximately 0.75–0.8 Å), indicating robust and stable performance across datasets. In contrast, CryoAtom and ModelAngelo show broader distributions with higher median values and longer tails, particularly noticeable on Testdata Set II. This suggests that while these tools can achieve accurate predictions, they exhibit greater variability, especially when processing more challenging density maps.

These results collectively demonstrate that Cα prediction performance is governed by a complex interplay among evaluation thresholds, dataset characteristics, and algorithmic design. ModelAngelo excels under lenient thresholds and on well-behaved maps, making it suitable for applications prioritizing backbone completeness. EModelX and DeepMainMast show strengths in high-precision regimes and maintain more conservative, stable predictions. The choice of evaluation criterion should align with specific application requirements: higher RMSD cutoffs (3 Å) better assess global backbone recovery, while lower cutoffs (1 Å) probe true atomic-level accuracy. For extended experimental details including comprehensive per-threshold metric distributions and additional statistical analyses, refer to Supplementary Material Section 2.2, available as supplementary data at *Bioinformatics* online, Fig. S3, available as supplementary data at *Bioinformatics* online, and Table 3, available as supplementary data at *Bioinformatics* online.

**Table 3:** Running time analysis on Testdata Set I dataset.^a^

Software	Size	Average time (minute)	*N*
CryoAtom	S	0.56	169
	M	0.71	174
	L	0.77	170
	**Total**	**0.68**	**513**
EModelX	S	1.00	169
	M	0.99	174
	L	1.62	170
	**Total**	**1.20**	**513**
DeepMainMast	S	29.80	169
	M	33.06	174
	L	54.78	170
	**Total**	**39.19**	**513**
ModelAngelo	S	1.54	169
	M	1.54	174
	L	2.91	170
	**Total**	**1.99**	**513**

a S=small, M=medium, L=large; N=count.

To complement this threshold-based benchmarking, we next disentangled model completeness from agreement to deposited references using a dedicated Cα-level analysis on Testdata Set II (*n* = 177), including a completeness index (Npr), representative case studies of unmodeled density, and Q-score-based density-evidence tests for unmatched predictions. Across methods, higher completeness was generally associated with more unmatched Cα atoms, whereas unmatched atoms showed variable density support depending on method and target. Full analyses are provided in Supplementary Material Section 3, available as supplementary data at *Bioinformatics* online.

#### 3.2.2 Speed and efficiency comparison

To evaluate the practical utility of the benchmarked tools, we analyzed the computational efficiency of four software packages on the Testdata Set I. The targets were stratified into three categories: small, medium, and large, based on the tertiles (33% and 66% quantiles) of their molecular weights. As summarized in Table 3, the comparative analysis reveals a clear stratification in throughput and scalability among the evaluated methods.

Conversely, EModelX and ModelAngelo showed a noticeable decline in efficiency for complex structures; for instance, ModelAngelo’s average runtime jumped from 1.54 min in the small-molecular-weight group to 2.91 min in the large-molecular-weight group. DeepMainMast was the most sensitive to molecular scale, with the average runtime surging by 84% (from 29.80 to 54.78 min) as complexity increased, reflecting the high computational complexity of its multi-task prediction architecture and intensive post-processing pipeline.

Collectively, these results underscore the impact of algorithmic design on structural modeling throughput. The substantial computational overhead associated with DeepMainMast is primarily attributable to its intricate multi-task prediction framework and intensive post-processing pipeline. In contrast, CryoAtom achieves superior efficiency through a streamlined network topology and an optimized clustering strategy. In practical applications, the selection of software necessitates a strategic balance between data volume and analytical precision. CryoAtom presents a clear temporal advantage for large-scale screening scenarios, whereas the moderate time investment required by EModelX and ModelAngelo is justified for complex structural analyses demanding high fidelity. Future research should prioritize the reduction of algorithmic complexity, with a specific focus on enhancing scalability for large-molecular-weight data.

### 3.3 Extended evaluation: impact of molecular weight, resolution, and SNR noise

#### 3.3.1 Scalability analysis across molecular weight scales

**Figure 3:**
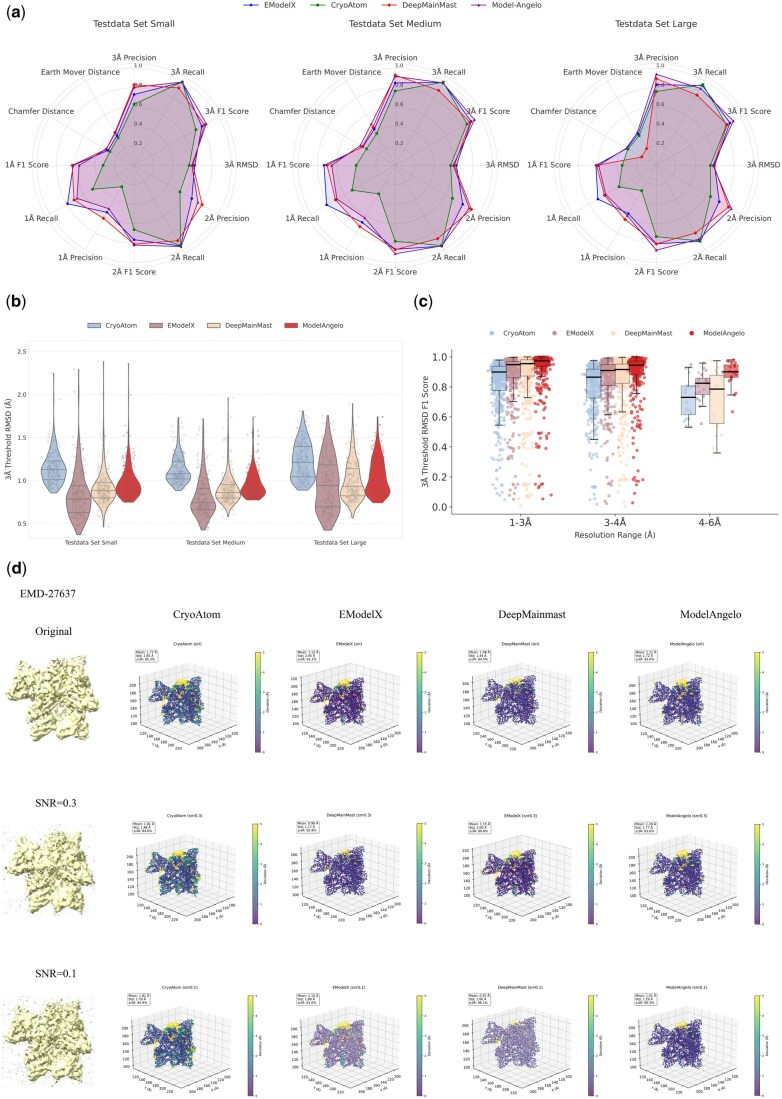
(a) Radar charts comparing precision, recall, F1-score, Chamfer Distance, and Earth Mover’s Distance for three molecular weight test datasets at 3, 2, and 1 Å RMSD thresholds. Colors represent different software tools. (b) Violin plots showing the Cα RMSD distribution of four software tools within the 3 Å RMSD threshold across three public test datasets. (c) Box plots with scatter points showing the F1-scores of four software tools at the 3 Å RMSD threshold across different resolution ranges. (d) Spatial distribution of local Cα prediction deviations under varying noise levels. A 3D heatmap matrix illustrates the spatial accuracy of Cα predictions for the four evaluated software tools (rows: ModelAngelo, DeepMainMast, EModelX, CryoAtom) across three signal-to-noise ratio conditions (columns: original, SNR = 0.3, SNR = 0.1). In each subplot, predicted Cα atoms are color-coded by their deviation distance to the nearest native Cα atom using a unified color scale (0–5 Å). Key statistical metrics are annotated: mean deviation (Mean Dev), standard deviation (SD), and the percentage of predictions within a 3 Å threshold (≤3 Å). This visualization reveals that while all methods demonstrate robustness to uniform Gaussian noise (with >84% of predictions within 3 Å even at SNR = 0.1), their error distribution patterns and noise sensitivity differ. For instance, DeepMainMast maintains the tightest cluster of low errors (lowest standard deviation) across noise levels, whereas CryoAtom exhibits more spatially dispersed deviations.

Molecular weight significantly modulates Cα prediction accuracy across all evaluated tools. As illustrated in the radar charts (Fig. 3a), performance stratification emerges clearly along both molecular scale and RMSD threshold dimensions (see Supplementary Material 2.3, available as supplementary data at *Bioinformatics* online, Fig. 4, available as supplementary data at *Bioinformatics* online for extended analysis). Under lenient 3 Å thresholds, all tools maintain reasonable completeness across molecular sizes, but precision-recall balance varies substantially. CryoAtom demonstrates near-perfect recall for small proteins but exhibits precision instability, suggesting over-prediction tendencies. ModelAngelo shows the most balanced performance for medium-sized proteins, maintaining stable F1-scores across different thresholds. DeepMainMast suffers significant performance degradation with large molecular weights, particularly under strict 1 Å thresholds where its recall plummets dramatically. In contrast, EModelX exhibits the highest adaptability with minimal performance fluctuations across diverse molecular scales.

The multi-dimensional assessment in Fig. 3a further elucidates the trade-offs between local accuracy and global topology. For small proteins, while DeepMainMast dominated in traditional detection metrics (as shown by the larger radar area), it yielded disproportionately high Chamfer Distance and Earth Mover’s Distance values. This discrepancy suggests that while individual atoms are identified, the overall topological similarity to the ground truth remains suboptimal. CryoAtom maintained stable distance metrics in the small and medium datasets but showed inconsistency in 1 Å threshold precision, indicating localized deviations in density fitting. In contrast, EModelX exhibited the most balanced radar profile across all datasets, suggesting its utility as a general-purpose modeler. As molecular weight increased to the large category, all tools showed a contraction in radar area, with DeepMainMast experiencing the most pronounced increases in distance metrics, reflecting its stronger comparative advantage in local prediction capability for large-molecular-weight proteins. Notably, CryoAtom and EModelX demonstrated greater resilience to threshold strictification, maintaining more consistent structural envelopes than their counterparts.

Analysis of the RMSD distributions within the 3 Å threshold (Fig. 3b) provides further insights into prediction consistency for medium and large molecular weight targets. EModelX demonstrates superior stability, with the tightest violin plot distributions, indicating its stronger capability in capturing local structural features. The other tools show broader RMSD distributions, suggesting greater variability in prediction accuracy for complex, high-molecular-weight systems.

In summary, Cα prediction performance is highly contingent upon molecular scale and RMSD thresholds. While all methods face challenges with increasing molecular weight, their responses differ significantly, highlighting the importance of selecting tools based on target size and the required balance between recall and precision.

#### 3.3.2 Resolution-dependent performance variations

Cryo-EM map resolution emerges as a primary determinant of Cα prediction accuracy, with performance stratification clearly observable across resolution regimes (Fig. 3c). At near-atomic resolutions (1–3 Å), all tools demonstrate robust performance with median F1-scores exceeding 0.8, indicating that high-quality density maps provide sufficient constraints for accurate backbone tracing. ModelAngelo achieves the highest median F1-score, underscoring its superior precision under optimal conditions. CryoAtom exhibits the greatest performance variability even in high-resolution scenarios. The transition to intermediate resolutions (3–4 Å) triggers a generalized decline in accuracy. ModelAngelo maintains relative superiority with a tempered decline, while CryoAtom shows particularly poor robustness with F1-score outliers approaching 0.4. In the low-resolution range (4–6 Å), all tools suffer significant reductions in performance, with median F1-scores falling below 0.4. ModelAngelo retains a marginal advantage, but DeepMainMast demonstrates clear unsuitability for low-resolution prediction through its extended distribution ranges.

Qualitative analysis of ultra-low resolution examples (6.9–8.2 Å) reveals distinct failure modes (Supplementary 2.4, available as supplementary data at *Bioinformatics* online, Fig. 5, available as supplementary data at *Bioinformatics* online) CryoAtom and EModelX capture global morphological contours but produce over-dense predictions with numerous false positives, while ModelAngelo and DeepMainMast generate fragmented, incomplete models with substantial atomic omissions.

These findings establish that resolution quality fundamentally constrains Cα prediction reliability. While high-resolution conditions enable accurate backbone tracing, performance degrades progressively with resolution deterioration, with different tools exhibiting characteristic failure patterns that should inform application-specific software selection.

#### 3.3.3 Evaluating SNR-dependent robustness

To rigorously assess this, we introduced varying levels of Gaussian noise (SNR = 0.3 and SNR = 0.1) into representative density maps. As shown in Fig. 3d, while all four tools demonstrated high robustness in predicting Cα positions without generating significant outliers–even with added noise–their individual performance levels varied. The spatial deviation heatmaps reveal nuanced differences: DeepMainMast exhibited the most consistent predictions (std ≈1.1 Å), while CryoAtom’s high-recall strategy resulted in more dispersed errors (std > 1.8 Å). Interestingly, ModelAngelo’s accuracy slightly improved with added noise (≤3 Å: from 93.0% to 96.3%), suggesting a potential regularization effect. This observation underscores the necessity of incorporating masking strategies during network training for Cα prediction to enhance noise resilience.

As SNR decreases from original maps to severely noisy conditions (SNR = 0.1), all tools experience structural fidelity loss, but ModelAngelo demonstrates exceptional resilience, maintaining the lowest Chamfer distance (4.665) and highest F1-score (0.924) under high-noise conditions (Supplementary 2.5, available as supplementary data at *Bioinformatics* online Fig. 6, available as supplementary data at *Bioinformatics* online). EModelX follows closely with stable performance across noise levels. In contrast, DeepMainMast adopts a conservative strategy characterized by high precision but severely reduced recall at low SNR (0.757), indicating a propensity to miss true atomic positions under noise interference. CryoAtom exhibits the opposite tendency, preserving high recall (0.968 at SNR = 0.3) but suffering from significantly lower precision (0.775), reflecting over-prediction susceptibility.

These findings reveal distinct algorithmic responses to noise: ModelAngelo and EModelX maintain balanced performance through noise-resilient feature extraction, while DeepMainMast and CryoAtom represent opposing ends of the sensitivity-specificity spectrum. Practical application selection should therefore consider both expected SNR conditions and the desired balance between atomic detection completeness and localization precision.

## 4 Discussion

Our systematic evaluation reveals that the performance of these four automated modeling tools is characterized by a universal balancing act between precision and recall and exhibits marked variability across diverse scenarios; we attribute these differentiating profiles to three key technical determinants: deep learning architecture, post-processing clustering strategy, and generalization to varied data distributions.

The deep learning backbone fundamentally shapes a method’s capability to extract and integrate features from cryo-EM density maps. As evidenced in Table 3, available as supplementary data at *Bioinformatics* online, ModelAngelo (FPN-based CNN) and DeepMainMast (UNet3+) consistently achieve the highest precision across datasets, particularly in the high-resolution Testdata Set I. This suggests that architectures emphasizing multi-scale feature fusion (FPN) or deep, dense connections (UNet3+) excel at learning discriminative local patterns, reducing false positives. However, this specialization can trade off robustness. DeepMainMast’s Chamfer and EMD distances degrade significantly in Testdata Sets II and III, indicating that its detailed, classification-focused output may be highly sensitive to resolution decay and noise patterns not prevalent in its training distribution. In contrast, EModelX (3D Residual U-Net) and CryoAtom (3D Convolutional U-Net), using more standardized encoder-decoder frameworks, demonstrate more consistent geometric metrics (CD, EMD) across diverse conditions. This points to a design trade-off: specialized architectures can achieve peak performance on ‘in-distribution’ data, while simpler, well-regularized networks may offer better generalization under data shift.

The post-processing step that condenses predicted density into discrete Cα positions is equally critical. The dichotomy between DBSCAN (used by EModelX) and MeanShift (used by the others) explains major trends in recall and specificity. Methods using MeanShift, particularly CryoAtom, achieve near-perfect recall (Table 3, available as supplementary data at *Bioinformatics* online), as this algorithm is adept at discovering density modes without a preset cluster number, favoring completeness. However, this can come at the cost of precision, as seen in CryoAtom’s lower precision scores. DBSCAN, by explicitly modeling noise and requiring a minimum density, produces more conservative, high-specificity clusters. This aligns with EModelX’s consistently high precision and superior RMSD, especially in high-resolution data where density is well-defined. The stark performance gap between ModelAngelo and CryoAtom-both using MeanShift but with different architectural front-ends-underscores that clustering operates on the features provided by the network; a noisy or poorly localized feature map will lead even a robust clusterer astray.

The progressive performance variation across Testdata Sets I, II, and III for all tools highlights the role of training data representativeness. Testdata Set III, dominated by large molecules and medium-resolution maps, presents a clear domain shift. Notably, DeepMainMast’s performance drops most precipitously here, suggesting its training data may have under-represented such challenging scenarios. Conversely, EModelX and ModelAngelo maintain relatively stable F1-scores, indicating better training on or inherent robustness to a broader data distribution. CryoAtom’s high recall persists but with lower precision, suggesting its training emphasized sensitivity over discriminative power for ambiguous regions. This analysis moves beyond simply reporting performance drops; it suggests that future benchmark designs must explicitly account for dataset bias and that method development should prioritize training strategies that enhance out-of-distribution robustness.

In summary, our results demonstrate that no single factor dominates; performance is a synergistic outcome of architecture, clustering, and data. Architectural choice sets the ceiling for feature quality, the clustering strategy determines how those features are translated into atomic coordinates, and the training data distribution defines the operational domain where these components are effective.

Moving forward, the development of automated Cα modeling tools should prioritize two interdependent directions: (i) creating adaptive neural architectures that are robust across a wider spectrum of data qualities, potentially through dynamic or attention mechanisms that weight features based on local map quality, and (ii) designing topology-aware clustering or optimization algorithms that incorporate structural priors (e.g. chain connectivity, residue spacing) to post-process network predictions, thereby enhancing geometric consistency. Integrating robust structural priors directly into the learning objective will be crucial for enhancing generalization. Ultimately, striking an optimal balance between representation learning, geometric constraints, and computational efficiency remains the key challenge for advancing the next generation of tools for structural biology.

## Supplementary Material

btag350_Supplementary_Data

## Data Availability

The code for the evaluation metrics is available at https://github.com/zhtianz/Benchmarking\_CA and supplementary data are available at Bioinformatics online.
