# Supplementary material for: Benchmarking deep learning methods for Cα atom prediction in cryo-EM density maps
Source: Bioinformatics. 2026 Jun 2;42(6):btag350. doi: 10.1093/bioinformatics/btag350 (PMC13264388; doi:10.1093/bioinformatics/btag350)
Supplement: btag350_Supplementary_Data [file btag350_supplementary_data.pdf]

## S1 DATASET DISTRIBUTION

### A. Benchmark Dataset

Our benchmark datasets collectively span a resolution range primarily between 2 to 4 Å, providing a comprehensive foundation for evaluating Cα atom prediction accuracy under diverse conditions Giri and Cheng (2024); Jamali et al. (2024). As shown in Figure S1, the three benchmark datasets exhibit complementary characteristics in molecular weight and resolution distributions. Testdata Set I provides balanced molecular size representation (40.2% small, 35.7% medium, 24.2% large) with predominantly high-resolution maps (70.2% <3 Å). Testdata Set II shows moderate enrichment of medium-weight molecules (39.5%) and medium-resolution maps (65.1% 3-5 Å). Testdata Set III presents a challenging test case with 82.0% large molecules and 96.1% medium-resolution maps. This progressive variation in dataset characteristics enables systematic evaluation of Cα prediction methods across diverse experimental conditions. The three benchmark datasets were integrated and subsequently partitioned based on the following criteria. The resulting data distribution is summarized in Table S1.

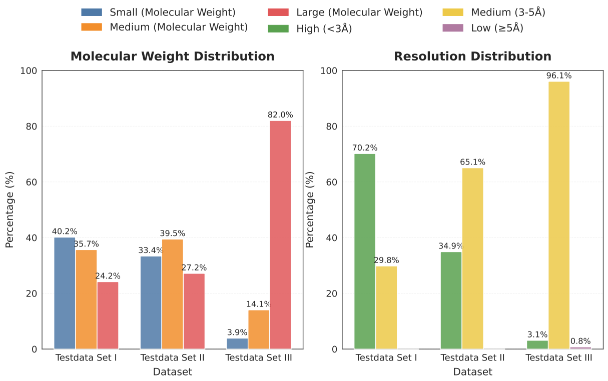


**Fig. S1.** Distribution of molecular weight (left) and resolution (right) in the benchmark datasets. Molecular weight is classified into Small, Medium, and Large categories based on Ca atom count percentiles. Resolution is grouped into High (<3 Å), Medium (3-5 Å), and Low (>=5 Å) ranges. The percentages represent sample proportions in each category.

**A. Resolution diversity** covering high, medium, and low-resolution ranges (1-3 Å, 3-4 Å, 4-6 Å, and >6 Å) to ensure comprehensive evaluation.

**B. Molecular weight variation** based on the native state PDB structures corresponding to each density map. We extracted Cα atoms from each PDB structure and categorized the density maps into large (molecular weight range: >=2280), medium (molecular weight range: 1166-2280), and small (molecular weight range: <1165) Cα atom prediction tasks to evaluate each software's performance in predicting Cα atoms across different molecular weights.

**C. Signal-to-noise ratio variation** by randomly selecting 48 density maps from the three datasets and adding noise with signal-to-noise ratios of 0.1 and 0.3 using the following strategy to evaluate the robustness of each software in Cα atom prediction.

**Table S1.** Statistical Summary of the cryo-EM Benchmark Dataset

| Category | Resolution Range (Å) | Count | Percentage (%) |
|---|---|---|---|
| **A. Resolution Distribution** | | | |
| High Resolution | 1.0-3.0 | 439 | 53.7 |
| Medium Resolution | 3.0-4.0 | 359 | 43.9 |
| Low Resolution | 4.0-6.0 | 20 | 2.4 |
| **Total** | **All** | **818** | **100.0** |
| **B. Molecular Weight Grouping (Based on the number of C$\alpha$)** | | | |
| Small Molecular Weight | $\leq$ 1165 | 270 | 33.0 |
| Medium Molecular Weight | 1166-2280 | 271 | 33.1 |
| Large Molecular Weight | $>$ 2280 | 277 | 33.9 |
| **C. Signal-to-noise ratio (SNR) Density Map Data Statistics** | | | |
| **C.1. Resolution Distribution** | | | |
| High Resolution | 1.0-3.0 | 22 | 45.8 |
| Medium Resolution | 3.0-4.0 | 25 | 52.1 |
| Low Resolution | 4.0-6.0 | 1 | 2.1 |
| **Subtotal** | **All** | **48** | **100.0** |
| **C.2. Molecular Weight Distribution (Based on the number of C$\alpha$)** | | | |
| Small Molecular Weight | $\leq$ 1135.6 | 16 | 33.3 |
| Medium Molecular Weight | 1135.6-1879.3 | 16 | 33.3 |
| Large Molecular Weight | $>$ 1879.3 | 16 | 33.3 |
| **Subtotal** | **All** | **48** | **100.0** |

*Note*: (A) Full dataset resolution distribution: 439 structures (53.7%) in 1.0-3.0 Å, 359 structures (43.9%) in 3.0-4.0 Å, and 20 structures (2.4%) in 4.0-6.0 Å. (B) Molecular weight grouping based on n_true values: 33rd percentile=1165.2, 66th percentile=2280.0. Small molecular weight (MW) group: $\leq$ 1165 atoms (n=270, 33.0%); Medium MW: 1166-2280 atoms (n=271, 33.1%); Large MW: $>$ 2280 atoms (n=277, 33.9%). (C) SNR density map subset (n=48) statistics: Resolution distribution: 22 structures (45.8%) in 1.0-3.0 Å, 25 structures (52.1%) in 3.0-4.0 Å, and 1 structure (2.1%) in 4.0-6.0 Å. Molecular weight grouping based on n_true values: 33rd percentile = 1135.6, 66th percentile=1879.3. Small MW group: $\leq$ 1135.6 atoms (n=16, 33.3%); Medium MW: 1135.6-1879.3 atoms (n=16, 33.3%); Large MW: $>$ 1879.3 atoms (n=16, 33.3%).

To enable a controlled analysis of the impact of resolution and signal-to-noise ratio, a standardized preprocessing protocol was uniformly applied. A signal-to-noise ratio (SNR)-based noise injection strategy was implemented to generate maps with precisely defined noise levels. This process involves first computing the signal power (variance) of the original density map. Subsequently, the requisite noise power is calculated based on the target SNR, and Gaussian noise of the corresponding magnitude is algorithmically generated and superimposed onto the original data. The mathematical formulation is given by:

$$\sigma_{\text{noise}} = \sqrt{\frac{\text{Var}(I)}{\text{SNR}_{\text{target}}}} \tag{S1}$$

Where $\text{Var}(I)$ is the variance of the original density map $I$, representing signal power. $\text{SNR}_{\text{target}}$ is the target signal-to-noise ratio value. $\sigma_{\text{noise}}$ is the standard deviation of the generated Gaussian noise. In practice, an original map is added to produce the noisy map. Critically, during the saving process, we preserved the header information of the original map file (unit cell dimensions, origin, and axis orientation) to ensure full compatibility with downstream analysis workflows. This rigorous preprocessing regimen guarantees that all evaluated software tools receive identical inputs, thereby ensuring the fairness and reproducibility of the comparative assessment.

## S2 EXPERIMENTS

### S2.1 How Critical is Accurate Cα Prediction for All-Atom Modeling?

This quantitative dependency is visually corroborated by case studies. For instance, in the prediction of EMD-22898 (PDB: 7kjr), substituting CryoAtom's predicted Cα trace with the native trace not only improved the overall fold but also corrected a false positive segment, resulting in a model more faithful to the ground truth (Figure S2, red box).

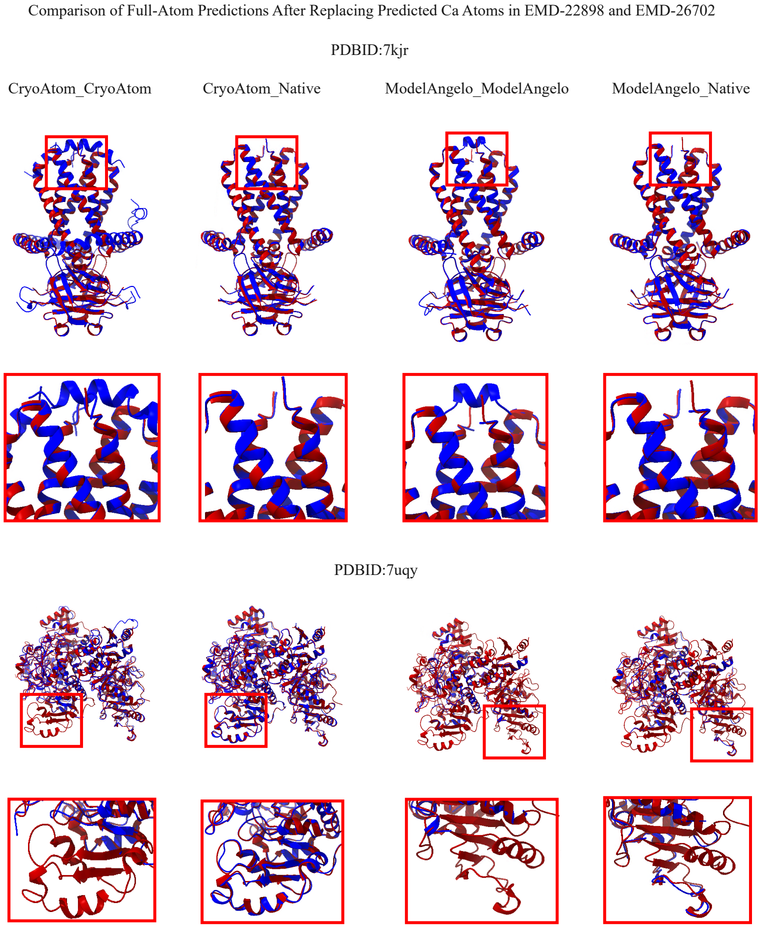


**Fig. S2.** Comparison of full-atom predictions after replacing Cα atoms in EMD-22898 and EMD-26702 using CryoAtom and ModelAngelo. The red structures represent the native state PDBs 7kjr (corresponding to EMD-22898) and 7uqy (corresponding to EMD-26702). The blue structures show the full-atom predictions from each software using either self-predicted Cα atoms. CryoAtom_CryoAtom indicates CryoAtom using its own predicted Cα atoms for full-atom prediction; CryoAtom_Native indicates CryoAtom using native Cα atoms; ModelAngelo_ModelAngelo indicates ModelAngelo using its own predicted Cα atoms; ModelAngelo_Native indicates ModelAngelo using native Cα atoms.

Table S2 data further indicates that full-atom models based on high-accuracy Cα atom predictions achieve higher precision. Specifically, high-accuracy native state Cα predictions improved CryoAtom's full-atom prediction TMscore by 0.0137 and ModelAngelo's by 0.0228. For the backbone Cα metric results, using native state Cα atoms ensures that their positions align perfectly with the ground truth. This result also demonstrates that the Cα atom trajectories in the full-atom predictions align with the software-predicted Cα trajectories. This further underscores the critical role of Cα atoms in full-atom prediction.

**Table S2.** Comparison of different methods for C$\alpha$ prediction on EMD-22898

| Method | Backbone C$\alpha$ Metrics (Precision/Recall/F1) | Backbone C$\alpha$ Distance Metrics (Chamfer/EMD/RMSD) | TMscore |
|---|---|---|---|
| CryoAtom_CryoAtom | 0.787/1.000/0.881 | 2.012/1.719/0.211 | 0.7270 |
| CryoAtom_Native | 1.000/1.000/1.000 | 0.255/0.127/0.166 | **0.7407** |
| ModelAngelo_ModelAngelo | 0.980/0.995/0.987 | 0.467/0.259/0.270 | 0.9766 |
| ModelAngelo_Native | 1.000/0.984/0.992 | 0.368/0.157/0.185 | **0.9994** |

### S2.2 Performance on benchmark datasets

Based on the evaluation results in Table S3, the four C$\alpha$ atom prediction software demonstrate distinct performance characteristics and adaptability across the three benchmark datasets. In

**Table S3.** Comparative evaluation of different software on three datasets. Metrics include Chamfer Distance (CD, Å), Earth Mover's Distance (EMD, Å), RMSD at 3 Å threshold (3 Å RMSD, Å), Precision, Recall, and F1-score at 3 Å threshold. Bold numbers indicate the smallest values for CD, EMD, and 3 Å RMSD, and the largest values for Precision, Recall, and F1-score.

**Testdata Set I**

| Software | CD | EMD | 3 Å RMSD | 3 Å Prec. | 3 Å Rec. | 3 Å F1 |
|---|---|---|---|---|---|---|
| CryoAtom | 6.512 | 4.310 | 1.105 | 0.7490 | **0.9888** | 0.8342 |
| EModelX | 4.414 | 2.947 | **0.772** | 0.8366 | 0.9849 | 0.8899 |
| DeepMainMast | 3.837 | 2.408 | 0.876 | **0.9175** | 0.9137 | 0.8951 |
| ModelAngelo | **3.640** | **2.203** | 0.934 | 0.9029 | 0.9806 | **0.9285** |

**Testdata Set II**

| Software | CD | EMD | 3 Å RMSD | 3 Å Prec. | 3 Å Rec. | 3 Å F1 |
|---|---|---|---|---|---|---|
| CryoAtom | 11.581 | 7.514 | 1.192 | 0.6563 | **0.9846** | 0.7525 |
| EModelX | 9.460 | 6.145 | **0.905** | 0.7246 | 0.9787 | 0.7975 |
| DeepMainMast | 60.269 | 26.203 | 0.960 | **0.8329** | 0.8580 | 0.7979 |
| ModelAngelo | **7.274** | **4.440** | 1.026 | 0.8135 | 0.9632 | **0.8500** |

**Testdata Set III**

| Software | CD | EMD | 3 Å RMSD | 3 Å Prec. | 3 Å Rec. | 3 Å F1 |
|---|---|---|---|---|---|---|
| CryoAtom | 7.502 | 6.609 | 1.365 | 0.6873 | **0.9411** | 0.7731 |
| EModelX | 7.331 | 5.780 | **1.151** | 0.7816 | 0.9243 | 0.8320 |
| DeepMainMast | 176.635 | 84.058 | 1.157 | 0.8204 | 0.7925 | 0.7695 |
| ModelAngelo | **7.070** | **5.099** | 1.186 | **0.9211** | 0.8805 | **0.8841** |

terms of distance-based metrics, ModelAngelo exhibits the most consistent geometric accuracy, achieving the lowest values (i.e., best performance) for both Chamfer Distance (CD) and Earth Mover's Distance (EMD) across all three datasets. Notably, it maintains relatively low distance errors even when processing the large-molecule dominated Testdata Set III (CD = 7.070 Å, EMD = 5.099 Å). In comparison, DeepMainMast performs excellently on Testdata Set I under high-resolution conditions with small molecules (CD = 3.837 Å, EMD = 2.408 Å), but its distance errors increase substantially on Testdata Sets II and III under large-molecule scenarios (Testdata Set III: CD = 176.635 Å, EMD = 84.058 Å), indicating its sensitivity to molecular size variations. EModelX achieves the best performance in 3 Å RMSD metric, obtaining the lowest values across all three

datasets (0.772-1.151 Å), demonstrating superior overall structural matching of predicted C$\alpha$ atom positions.

Regarding threshold-based metrics, each software shows different strengths across datasets. DeepMainMast achieves the highest precision on Testdata Set I under high-resolution conditions (0.9175), while ModelAngelo demonstrates optimal precision on Testdata Set III under medium-resolution large-molecule conditions (0.9211). CryoAtom excels particularly in recall, achieving the highest values on Testdata Sets I and II (>0.984), indicating its capability to detect the highest proportion of true C$\alpha$ atoms. Comprehensive F1 scores reveal that ModelAngelo performs best on Testdata Sets I (0.9285) and II (0.8500), while EModelX slightly outperforms ModelAngelo on Testdata Set III (0.8320 vs. 0.8841).

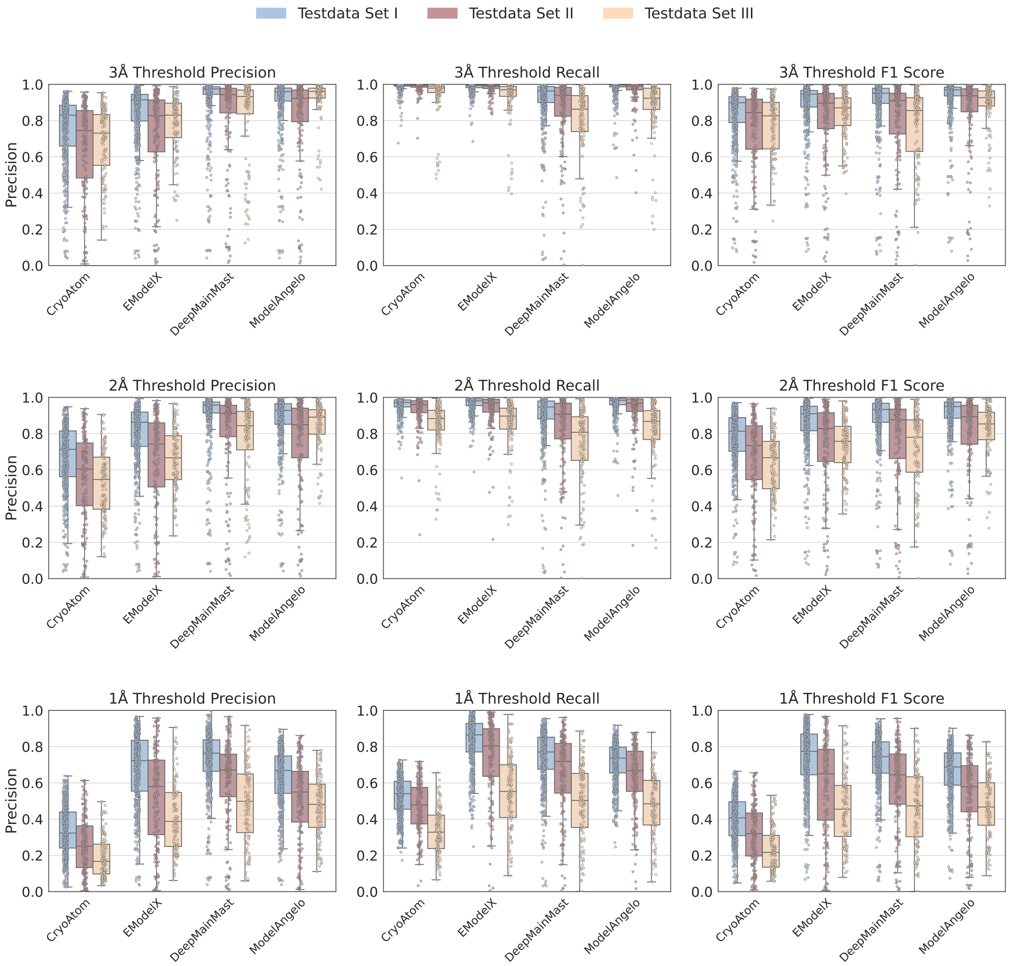


**Fig. S3.** Box plots with scatter points showing precision, recall, and F1-score at 3 Å, 2 Å, and 1 Å RMSD thresholds. The x-axis represents the software tools: CryoAtom, EModelX, DeepMainMast, and ModelAngelo. The y-axis shows the metric scores. Datasets are colored: blue for Testdata Set I, red for Testdata Set II and yellow for Testdata Set III.

Our analysis revealed a clear performance stratification among the programs, heavily influenced by the evaluation threshold and dataset characteristics. As illustrated by the precision-recall analysis across thresholds (Figure S3, a classic trade-off was observed. Stricter thresholds (e.g., 1 Å) universally lowered both precision and recall, testing the limits of atomic-level accuracy. Conversely, a more lenient 3 Å threshold allowed for higher completeness, better reflecting the overall success in capturing the protein backbone fold. at the lenient threshold of 3 Å, CryoAtom achieves a recall rate close to 1.0 (particularly on the Testdata Set III), indicating that it captures nearly all C$\alpha$ atoms, albeit with relatively lower precision, consistent with a tendency toward over-prediction. ModelAngelo maintains a more balanced precision–recall, with stable F1 scores

datasets, reflecting a conservative yet reliable prediction strategy. When the threshold is tightened to 1 Å, CryoAtom's metrics drop significantly (recall falls below 0.5), while ModelAngelo shows a smaller loss in precision but a notable decrease in recall, indicating that ModelAngelo prioritizes localization accuracy at the cost of missing some true atoms. EModelX and DeepMainMast exhibit intermediate behavior; DeepMainMast shows a relatively slower decline in F1-score at 1 Å, suggesting somewhat better resilience under stringent localization requirements. Dataset effects are also evident: Testdata Set I yields the broadest spread of metrics (e.g., CryoAtom's precision ranging from 0.4 to 1.0 Å), consistent with its challenging samples and heterogeneous samples. By contrast, the Testdata Set III yields more concentrated and generally higher scores, suggesting near-ideal conditions, whereas the Testdata Set II shows pronounced tool-dependent differences, such as the particularly strong performance of ModelAngelo.

Collectively, these benchmark results show that C$\alpha$ prediction performance is highly sensitive to both the chosen accuracy criterion (RMSD threshold) and dataset characteristics. ModelAngelo and CryoAtom excel under lenient thresholds and idealized or well behaved maps but become less reliable under stringent atomic level cutoffs. EModelX and DeepMainMast exhibit strong potential in high precision regimes (1 Å) and maintain more conservative, stable predictions, but would benefit from improved robustness at very high recall. Therefore, in practical applications, method selection should be aligned to specific analysis goals: ModelAngelo is particularly suitable when high recall and rapid backbone screening are prioritized, whereas EModelX or DeepMainMast may be preferable when localization accuracy is critical for detailed structural interpretation. Furthermore, the choice of benchmark datasets is crucial: Testdata Set I better probes generalization to realistic, heterogeneous maps, while Testdata Set III and similar idealized datasets may overestimate performance and obscure weaknesses in challenging, low signal to noise scenarios.

### S2.3 Performance for different molecular weights

As illustrated in Figure S4, modeling efficacy is intricately coupled with both molecular scale and the imposed RMSD thresholds. Under a permissive threshold (3 Å), all programs achieved generally high precision and recall, yet distinct performance stratification emerged across molecular weight categories. On the small molecular weight dataset, CryoAtom achieved a near-perfect recall ($\sim$ 1.0), though its precision exhibited substantial variance (noted by the expanded interquartile ranges), suggesting a tendency toward over-prediction or instability in low-complexity regions. ModelAngelo demonstrated superior equilibrium between precision and F1-score on medium-scale structures, yielding higher median values that underscore its reliability for typical protein assemblies. DeepMainMast showed significant performance degradation on large molecular weight datasets; its recall plummeted under strict thresholds (1 Å), revealing limited robustness when navigating the expanded search space of complex macromolecular architectures. While all methods experienced a decline in metrics at the 1 Å level, EModelX maintained the highest adaptability, characterized by minimal performance fluctuations across diverse scales.

In summary, C$\alpha$ prediction performance is highly contingent upon molecular scale and RMSD thresholds. ModelAngelo and CryoAtom performs best overall under small molecular weight and loose thresholds, but with poorer structural similarity metrics and significant performance degradation as molecular weight increases; EModelX shows prominent advantages in localization accuracy under medium molecular weight and strict thresholds, but requires improvement in local consistency; ModelAngelo exhibits balanced performance with small fluctuations across different conditions, suitable for generalized applications; Although DeepMainMast performs less robustly in high-molecular-weight scenarios, it possesses a certain capability for capturing local features. In practical applications, if processing small and medium proteins with prioritizing recall, ModelAngelo and EModelX is the optimal choice; if focusing on precise localization of medium-scale structures, EModelX is more reliable; for scenarios requiring balanced performance, EModelX is a safe option.

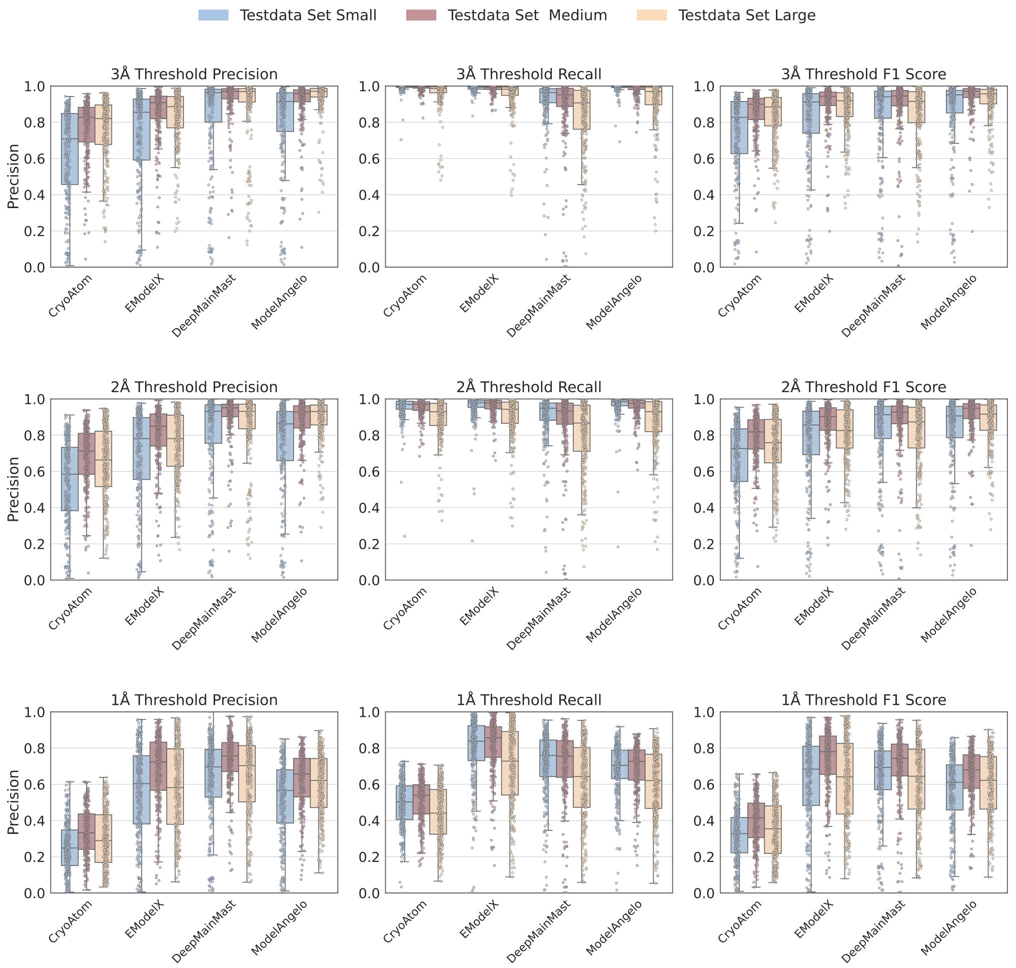


**Fig. S4.** Box plots with scatter points showing precision, recall, and F1-score at 3 Å, 2 Å, and 1 Å RMSD thresholds. The x-axis represents the software tools: CryoAtom, EModelX, DeepMainMast, and ModelAngelo. The y-axis shows the metric scores. Colors indicate molecular weight: blue for Testdata Set Small molecular weight, red for Testdata Set Medium molecular weight, and yellow for Testdata Set Large molecular weight.

### S2.4 Performance at low resolutions

The qualitative visualization of C$\alpha$ prediction at low resolutions (6.9 Å and 8.2 Å) in Figure S5 (utilizing EMD-6284 and EMD-6207), provides an intuitive comparison of the four programs. Using the native C$\alpha$ coordinates as a benchmark (red), we observe distinct failure modes across the predictors (blue). Overall, structural recognition becomes increasingly fraught beyond 6 Å, though clear stratification emerges. CryoAtom and EModelX successfully capture the global morphological contours of the density maps; however, they struggle with interaction fidelity, frequently misidentifying noise-induced artifacts as C$\alpha$ atoms. This results in an over-predicted, dense point cloud that obscures the true sparse architecture of EMD-6284 and EMD-6207. Conversely, ModelAngelo and DeepMainMast, likely constrained by the scarcity of low-resolution samples in their training distributions, produce highly fragmented models with substantial atomic omissions. These tools fail to recognize basic structural motifs under severe detail blurring, leading to sparse and incomplete predictions.

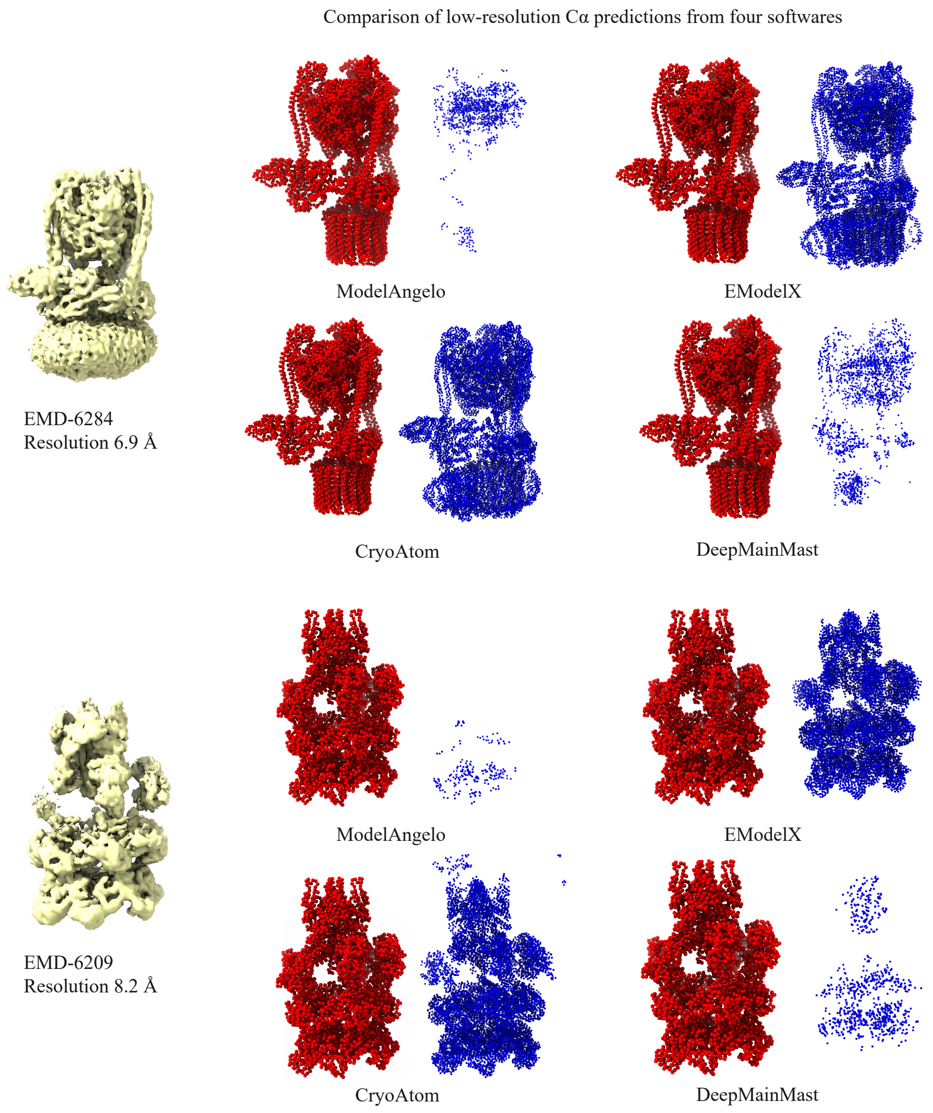


**Fig. S5.** Comparison of low-resolution C$\alpha$ predictions from CryoAtom, EModelX, DeepMainMast, and ModelAngelo against ground truth. Using EMD-6284 (Resolution 6.9 Å) and EMD-6209 (Resolution 8.2 Å) as examples, red points represent C$\alpha$ atoms extracted from the native state PDB, while blue points represent C$\alpha$ atoms predicted by the four software tools.

In summary, the transition from high (1-3 Å) to low (4-6 Å) resolution triggers a progressive decline in F1-scores and heightened stochastic fluctuations across all methods. ModelAngelo exhibits relative dominance across tiers but suffers from diminished stability at the lower limit. CryoAtom demonstrates commendable robustness in the intermediate-to-low range, while EModelX and DeepMainMast prove highly sensitive to signal-to-noise degradation. Notably, while CryoAtom and EModelX maintain the ability to delineate density contours, all four tools exhibit a propensity for false-positive noise assignments, which significantly escalates computational overhead and processing time.

### S2.5 Evaluating SNR-Dependent Robustness

Signal-to-Noise Ratio (SNR) significantly influences C$\alpha$ prediction robustness, with performance degradation patterns varying systematically among the evaluated tools (Table S4).

**Table S4.** Performance metrics at different SNR levels

| Software | SNR | Distance Metrics | | 3 Å rmsd Metrics | | |
|---|---|---|---|---|---|---|
| | | Chamfer | EMD | Precision | Recall | F1 |
| CryoAtom | 0.1 | 5.794 | 3.753 | 0.774 | 0.965 | 0.852 |
| | 0.3 | 5.737 | 3.804 | 0.775 | 0.968 | 0.854 |
| | original | **4.158** | **2.759** | **0.792** | **0.990** | **0.872** |
| EModelX | 0.1 | 4.338 | 2.741 | 0.848 | 0.958 | 0.896 |
| | 0.3 | 4.286 | 2.764 | 0.850 | 0.962 | 0.899 |
| | original | **2.670** | **1.754** | **0.874** | **0.984** | **0.922** |
| DeepMainMast | 0.1 | 6.172 | 4.520 | 0.915 | 0.757 | 0.801 |
| | 0.3 | 5.421 | 3.830 | 0.913 | 0.846 | 0.864 |
| | original | **3.180** | **2.235** | **0.933** | **0.900** | **0.904** |
| ModelAngelo | 0.1 | 4.665 | 3.125 | 0.932 | 0.926 | 0.924 |
| | 0.3 | 4.319 | 2.797 | 0.922 | 0.948 | 0.932 |
| | original | **2.657** | **1.697** | **0.930** | **0.978** | **0.951** |

## S3 COMPLETENESS ANALYSIS AND DENSITY-EVIDENCE ASSESSMENT FOR THE C$\alpha$ BENCHMARK

Our primary benchmark evaluates C$\alpha$ localization against deposited reference structures. While this design isolates front-end backbone tracing accuracy, it may underrepresent methods that favor completeness and predict additional residues in density regions not present in deposited models. Therefore, we provide complementary analyses to separate completeness from precision and to assess density evidence for additionally modeled features. We conducted the following experiments and analyses on Testdata Set II, which contains 177 test cases.

### S3.1 Completeness-Oriented Metric (Npr)

To further examine the completeness tendency of each method, we introduced a prediction completeness index, Npr, defined as the ratio of the number of predicted C$\alpha$ atoms to the number of C$\alpha$ atoms in the reference structure (Npr = Npred/Nref). Here, the reference structure is defined as the deposited native PDB model paired with each EMDB map in the benchmark dataset. This metric was summarized in Table S5 and Figure S6.

**Table S5.** Descriptive statistics of Npr for each method

| Method | Mean | Median | Q1 | Q3 |
|---|---|---|---|---|
| CryoAtom | 4.0725 | 1.6764 | 1.4119 | 2.4840 |
| EModelX | 3.0981 | 1.2835 | 1.1200 | 1.8167 |
| DeepMainMast | 2.0264 | 1.0101 | 0.9046 | 1.1277 |
| ModelAngelo | 2.3901 | 1.1367 | 1.0386 | 1.4162 |

As shown in Table S5, CryoAtom and EModelX yielded larger Npr values (median Npr = 1.6764 and 1.2835, respectively), indicating a stronger tendency to produce more complete models than the reference structure. In contrast, DeepMainMast produced predictions closest to the reference completeness (median Npr = 1.0101), while ModelAngelo showed an intermediate

behavior (median Npr = 1.1367). The corresponding boxplot of $\log_{10}(\text{Npr})$ further illustrates the distribution and variability of completeness across methods.

Importantly, Npr reflects completeness only and does not assess whether extra predicted residues are supported by density evidence. Therefore, we interpret Npr together with the main accuracy-related metrics, including Chamfer Distance (CD), Earth Mover's Distance (EMD), Recall, Precision, and F1-score, to provide a more balanced characterization of model-building performance.

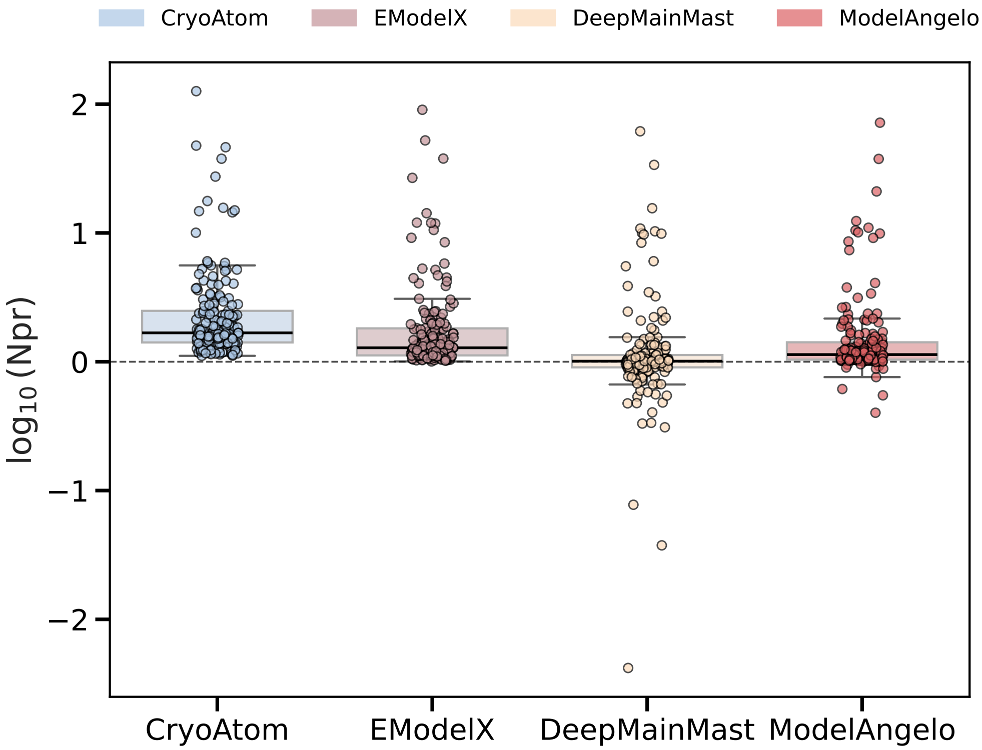


**Fig. S6.** Distribution of the prediction completeness index (Npr) across methods on Testdata Set II (177 test cases). The Npr value is defined as Npred/Nref, where Npred is the number of predicted C$\alpha$ atoms and Nref is the number of C$\alpha$ atoms in the reference structure, i.e., the deposited native PDB model corresponding to each EMDB map in the benchmark. Values are visualized using a boxplot of $\log_{10}(\text{Npr})$ to improve readability across the wide dynamic range. Individual test cases are shown as points.

### S3.2 Case studies in incompletely modeled density regions

To test whether additional predictions can be density-supported, we examined two representative targets with apparent unmodeled map regions: EMD-27253/PDB: 8d8o and EMD-33678/PDB: 8y82 (Fig. S7). For each method, we report total predicted C$\alpha$ atoms (Npred), unmatched counts under a 3.0 Å criterion, and median Q-score of unmatched atoms.

For Fig. S7a, EMD-27253 (PDB: 8d8o; Resolution: 3.35 Å), the deposited model leaves a visible density region unmodeled. CryoAtom and EModelX expanded most strongly into unmodeled density, ModelAngelo shows partial extension, and DeepMainMast remains comparatively conservative with a larger uncovered area. Quantitatively, CryoAtom produced the largest prediction set (Npred = 4169; unmatched = 2241; unmatched median Q-score = 0.32), consistent with strong completeness tendency but also more low-confidence additions. EModelX (Npred = 2735; unmatched = 356; unmatched median = 0.39) and ModelAngelo (Npred = 2117; unmatched = 444; unmatched median = 0.39) were more conservative than CryoAtom. DeepMainMast predicted the fewest atoms (Npred = 1152; unmatched = 30; unmatched median = 0.63), indicating high local support for the small unmatched set but reduced coverage in this example.

For Fig. S7b, EMD-33678 (PDB: 8y82; Resolution: 2.83 Å), all four methods extended into

reference-unmodeled density and largely covered the region; unmatched predictions had higher Q-scores than in EMD-27253 (unmatched Q-score medians: CryoAtom 0.50, DeepMainMast 0.59, EModelX 0.57, ModelAngelo 0.495), suggesting that a substantial fraction of additional residues may correspond to density-supported features rather than purely spurious predictions.

These examples indicate that some predictions counted as false positives under deposited-structure matching can still be map-supported.

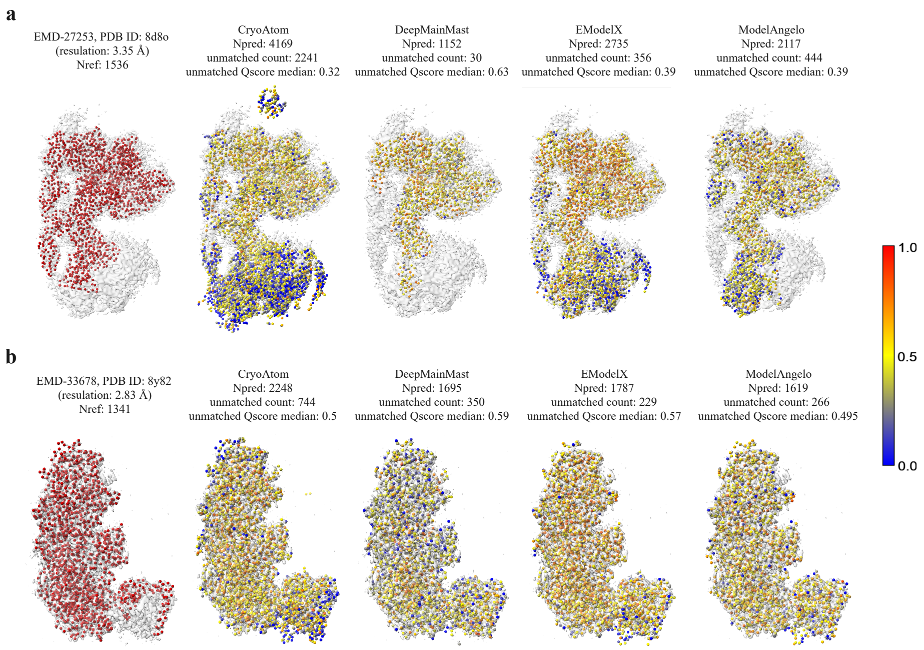


**Fig. S7. Case studies in incompletely modeled or previously unassigned density regions. a**, EMD-27253 (3.35 Å), PDB 8d8o; **b**, EMD-33678 (2.83 Å), PDB 8y82. For each case, the leftmost panel shows deposited-reference C$\alpha$ atoms (red) overlaid on the density map (gray). The four right panels show C$\alpha$ predictions from CryoAtom, DeepMainMast, EModelX and ModelAngelo, respectively, on the same map. Predicted atoms are colored by per-atom Q-score using a blue-yellow-red palette, displayed on a 0-1 scale (values $< 0$ and $> 1$ are clipped to the corresponding bounds for visualization). Text above each method panel reports total predicted C$\alpha$ count (Npred), unmatched C$\alpha$ count under the 3.0 Å matching criterion, and median Q-score of unmatched C$\alpha$ atoms.

### S3.3 Density-evidence assessment of additionally modeled C$\alpha$ features

We next evaluated map support globally using Q-scores Pintilie et al. (2020) for matched versus unmatched predictions (3.0 Å nearest-neighbor criterion to deposited C$\alpha$; on Testdata Set II, $n = 177$ targets). Per-target matched–unmatched differences were tested using paired Wilcoxon signed-rank tests.

As shown in Table S6 and Figure S8, CryoAtom, EModelX and ModelAngelo had higher Q-scores in matched than unmatched groups (for example, matched vs unmatched means: 0.4804 vs 0.3986; 0.6174 vs 0.4746; 0.5046 vs 0.4272, respectively), indicating weaker average density support among unmatched atoms. DeepMainMast showed the opposite trend (0.4351 vs 0.4867), suggesting that part of its unmatched set is still density-supported and may correspond to features absent from deposited models.

The matched–unmatched differences are statistically significant for all four methods (paired Wilcoxon signed-rank test, all $p < 10^{-15}$; Table S6). These findings indicate that distance-based matching to deposited structures alone is insufficient to fully characterize additional modeled residues. Integrating Q-score evidence provides a more informative interpretation of unmatched predictions, particularly when deposited models are incomplete.

**Table S6.** Summary statistics of 3.0 Å matched and unmatched Cα Q-scores for each method on Testdata Set II.

| Method | Matched | | Unmatched | | $p$-value | Unmatched count |
|---|---|---|---|---|---|---|
| | Mean | Median | Mean | Median | | |
| CryoAtom | 0.4804 | 0.4884 | 0.3976 | 0.3994 | $1.01 \times 10^{-29}$ | 411481 |
| EModelX | 0.6174 | 0.6358 | 0.4746 | 0.4820 | $1.76 \times 10^{-30}$ | 291648 |
| DeepMainMast | 0.4351 | 0.4319 | 0.4867 | 0.4900 | $7.39 \times 10^{-16}$ | 104537 |
| ModelAngelo | 0.5046 | 0.5141 | 0.4272 | 0.4380 | $1.85 \times 10^{-27}$ | 146263 |

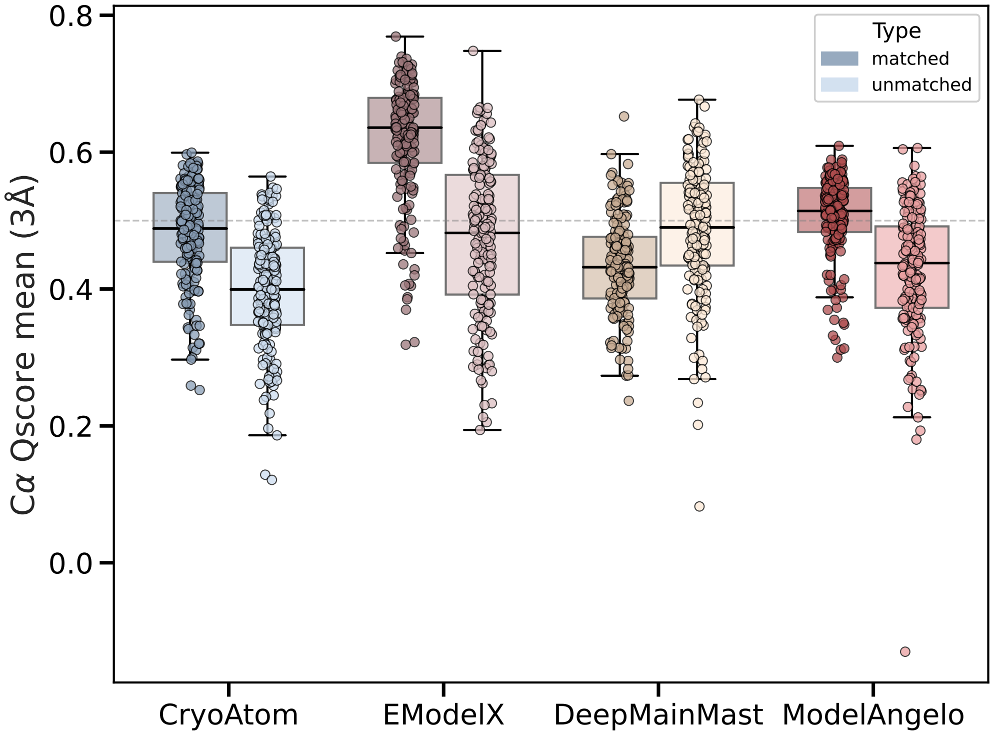


**Fig. S8.** Distribution of per-target mean Cα Q-scores at the 3.0 Å matching threshold for CryoAtom, EModelX, DeepMainMast, and ModelAngelo. Darker boxes indicate matched predictions and lighter boxes indicate unmatched predictions. Each point represents one target ($n = 177$). Boxes show the interquartile range (IQR), and center lines indicate medians.

## S4 BENCHMARKING EXECUTION PROTOCOL

To ensure reproducibility and a fair comparison, all evaluated software tools were executed by strictly following their official documentation and using default parameters unless otherwise specified. Below we detail the standardized input data, execution workflow, and tool-specific configurations.

### Input Data

For each test case, the input consisted of: (1) a cryo-EM density map in MAP/MRC format, and (2) the corresponding protein sequence(s) in FASTA format, as deposited in the PDB. The nominal global resolution of each map, as reported in the EMDB entry, was used as metadata but was not provided as a direct numerical parameter to any tool. For methods that require a contour level as input (e.g., DeepMainMast), we consistently used the **recommended contour level** provided in the corresponding EMDB map metadata to define the molecular boundary. This ensures the evaluation reflects performance under recommended usage conditions.

### Execution and Parameter Policy

Each tool was run in a dedicated conda environment as specified in its official repository. We used the latest stable version available at the time of the study. The standard command-line interfaces were used with all non-essential post-processing and refinement steps disabled to isolate the C$\alpha$ prediction module. This allows for a direct comparison of the core atomic localization capability. The exact commands and any necessary configuration file modifications are documented in the following subsections.

### Specific Consideration for ModelAngelo Modes

ModelAngelo offers two primary modeling modes: `build` (with sequence) and `build_no_seq` (without sequence). It is important to clarify that, for the benchmark target of **first-stage C$\alpha$ coordinate prediction**, the sequence information does not alter the initial C$\alpha$ candidate generation. The neural network that predicts C$\alpha$ density and performs initial clustering is identical in both modes. The performance differences between the modes manifest in subsequent steps involving sequence assignment and GNN-based refinement, which are beyond the scope of this coordinate-level evaluation. Therefore, to benchmark the core localization algorithm, we extracted C$\alpha$ atoms from the intermediate output generated before the first refinement round, which is common to both pipelines. This ensures our evaluation focuses purely on spatial accuracy, not on downstream sequence modeling.

### Detailed Software Configuration and Execution

Table S7 provides a concise summary of the execution configuration for each method. The following paragraphs detail the specific steps and command-line arguments used to generate the C$\alpha$ predictions for evaluation.

#### *ModelAngelo Execution Details*

**Configuration Modification:** To isolate the first-stage C$\alpha$ prediction, the parameter `"num_rounds":3` in the configuration file (`nucleotides_no_seq/config.json`) was changed to `"num_rounds":0`.
**Execution Command:**

```
conda activate model_angelo
model_angelo build_no_seq -v [map_path] -o [output_path]
      --keep-intermediate-results
```

The C$\alpha$ coordinates for evaluation were then extracted from the file:
`${output_path}/see_alpha_output/see_alpha_output_ca.cif`.

#### *CryoAtom Execution Details*

**Configuration Modification:** Similar to ModelAngelo, the parameter `"num_rounds":3` in the configuration file (`CryoAtom/config.json`) was set to `"num_rounds":0` to disable iterative modeling.
**Execution Command:**

```
conda activate CryoAtom
cryoatom build --map-path [map_path] --sequence-path [fasta_path]
      --output-dir [output_path] --keep-intermediate-results
```

The C$\alpha$ coordinates for evaluation were extracted from: `${output_path}/see_alpha_output/see_alpha_output_ca.cif`.

**Table S7.** Concise summary of software configurations for C$\alpha$ prediction benchmark.

| **Method** | **ModelAngelo v1.0.0** |
|---|---|
| Repository | https://github.com/3dem/model-angelo |
| Primary Inputs | Cryo-EM map (.map) |
| Key Configuration | num_rounds: 0 (disable iterative modeling) |
| C$\alpha$ Output File | <output>/see_alpha_output/see_alpha_output_ca.cif |
| **Method** | **CryoAtom v2.0.0** |
| Repository | https://github.com/YangLab-SDU/CryoAtom |
| Primary Inputs | Cryo-EM map (.map), FASTA sequence |
| Key Configuration | num_rounds: 0 (disable iterative modeling) |
| C$\alpha$ Output File | <output>/see_alpha_output/see_alpha_output_ca.cif |
| **Method** | **DeepMainMast v1.0.0** |
| Repository | https://github.com/kiharalab/DeepMainMast |
| Primary Inputs | Cryo-EM map (.map), FASTA sequence, Contour level |
| Key Configuration | Pipeline truncated after C$\alpha$ clustering. Contour level set to EMDB recommended value. |
| C$\alpha$ Output File | <output>/results/NODE_p0.3.pdb |
| **Method** | **EModelX v1.0** |
| Repository | https://github.com/biomed-AI/EModelX |
| Primary Inputs | Cryo-EM map (.map), FASTA sequence |
| Key Configuration | C$\alpha$ coordinates extracted directly from the point cloud after the internal clustering step. |
| C$\alpha$ Output File | Extracted coordinates saved to a new '.cif' file. |

#### *DeepMainMast Execution Details*

**Execution Command:** The provided multi-threaded script was executed, and the pipeline was manually truncated after the C$\alpha$ clustering step to obtain the initial prediction.

```
conda activate deepmainmast
./dmm_full_multithreads.sh -p [program_path] -m [map_path]
      -f [fasta_path] -c [contour] -o [output_path]
      -t [path_training_time] -T [fragment_assembling_time]
      -C [num_cpu] -M [num_cpu]
```

The C$\alpha$ coordinates for evaluation were taken from: `${output_path}/results/NODE_p0.3.pdb`.

#### *EModelX Execution Details*

**Execution Command:** The standard execution command was used.

```
conda activate EModelX
python run.py --protocol=temp_free --EM_map=[map_path]
      --fasta=[fasta_path] --output_dir=[output_path]
      --run_pulchra --pulchra_path modules/pulchra304/src/pulchra
```

The C$\alpha$ coordinates were extracted programmatically from the point set generated immediately after the internal `clustering()` function and saved into a new CIF file for evaluation.

### Fair Comparison Principle

To ensure a fair comparison across methods, all software packages were executed by strictly following the official instructions in their corresponding GitHub README documentation. Unless otherwise stated, we used default parameters and did not perform method-specific hyperparameter tuning. In this benchmark, we evaluate only first-stage C$\alpha$ localization accuracy at the point-cloud level. For each method, the evaluated output is the C$\alpha$ coordinate set directly produced by running the method with the standardized commands described above. Evaluation was performed by geometric matching between predicted and reference C$\alpha$ coordinates. No

amino-acid identity, residue type, or sequence alignment information was used in the scoring stage. Therefore, all reported metrics reflect coordinate-level C$\alpha$ localization performance rather than sequence-assignment performance.

## REFERENCES

Giri, N. and J. Cheng (2024). De novo atomic protein structure modeling for cryoem density maps using 3d transformer and hmm. *Nature Communications 15*(1), 5511.

Jamali, K. et al. (2024). Automated model building and protein identification in cryo-em maps. *Nature 628*(8007), 450–457.

Pintilie, G., K. Zhang, Z. Su, et al. (2020). Measurement of atom resolvability in cryo-em maps with q-scores. *Nature Methods 17*, 328–334.
